# Brain structure alterations in girls with central precocious puberty

**DOI:** 10.3389/fnins.2023.1215492

**Published:** 2023-07-20

**Authors:** Shoko Yoshii, Tomozumi Takatani, Tadashi Shiohama, Rieko Takatani, Yutaka Konda, Shinya Hattori, Hajime Yokota, Hiromichi Hamada

**Affiliations:** ^1^Department of Pediatrics, Graduate School of Medicine, Chiba University, Chiba, Japan; ^2^Center for Preventive Medical Sciences, Chiba University, Chiba, Japan; ^3^Department of Radiology, Chiba University Hospital, Chiba, Japan; ^4^Department of Diagnostic Radiology and Radiation Oncology, Graduate School of Medicine, Chiba University, Chiba, Japan

**Keywords:** central precocious puberty, precuneus cortex, voxel-based morphometry, surface-based morphometry, brain magnetic resonance imaging

## Abstract

**Purpose:**

Central precocious puberty (CPP) is puberty that occurs at an unusually early age with several negative psychological outcomes. There is a paucity of data on the morphological characteristics of the brain in CPP. This study aimed to determine the structural differences in the brain of patients with CPP.

**Methods:**

We performed voxel- and surface-based morphometric analyses of 1.5 T T1-weighted brain images scanned from 15 girls with CPP and 13 age-matched non-CPP controls (NC). All patients with CPP were diagnosed by gonadotropin-releasing hormone (GnRH) stimulation test. The magnetic resonance imaging (MRI) data were evaluated using Levene’s test for equality of variances and a two-tailed unpaired t-test for equality of means. False discovery rate correction for multiple comparisons was applied using the Benjamini–Hochberg procedure.

**Results:**

Morphometric analyses of the brain scans identified 33 candidate measurements. Subsequently, increased thickness of the right precuneus was identified in the patients with CPP using general linear models and visualizations of cortical thickness with a t-statistical map and a random field theory map.

**Conclusion:**

The brain scans of the patients with CPP showed specific morphological differences to those of the control. The features of brain morphology in CPP identified in this study could contribute to further understanding the association between CPP and detrimental psychological outcomes.

## 1. Introduction

Puberty is the onset of reproductive capability in humans accompanied by the development of secondary sexual characteristics and accelerated bone maturation. Central precocious puberty (CPP) is puberty that occurs at an unusually early age. Unusually early is defined for CPP diagnostic purposes as earlier than 8 years of age in girls and earlier than 9 years of age in boys (Bradley et al., 2020). It is usually idiopathic and is more prevalent in girls (Rosenfield et al., 2000). The prevalence of CPP was 193.2 per 100,000 persons (girls, 410.6; boys, 10.9) in an Asian country (Kim et al., 2019). In patients with CPP, levels of follicle-stimulating hormone (FSH), luteinizing hormone (LH), and sex hormones dramatically increase at an early age.

Although the increase of these hormones is the same as that during the normal process of puberty, timing of the increase of these hormones is inappropriately early, which is a distinct condition in CPP. CPP has several adverse effects, such as shorter final height as a result of early bone maturation, having difficulties in social life and psychosocial/behavioral sequelae due to an early menarche (De Sanctis et al., 2019). Moreover, CPP has been associated with several psychological problems that can have significant long-term impacts. These problems include depression, substance abuse, eating disorders, body dissatisfaction, externalizing behavior, risky sexual behavior, abortion, and certain aspects of academic achievement (Udry, 1979; Graber et al., 1997; Stice et al., 2001; Graber et al., 2004). Given the significant impact that these psychological problems can have on an individual’s subsequent life, it is essential to understand the causal relationships and mechanisms underlying CPP and its associated negative outcomes (Soriano-Guillen and Argente, 2019). While intracranial tumors are involved in some CPP cases, most CPP cases showed no abnormalities on MRI imaging as qualitative image analysis (Cantas-Orsdemir et al., 2018). Regarding quantitative analysis, several reports suggest that pituitary volume is significantly higher in CPP patients than in age-matched non-CPP controls (NC; Sharafuddin et al., 1994; Wu et al., 2020) as the pituitary gland is a hormone-producing site. However, pituitary changes are unlikely to be directly related to psychiatric symptoms because it is not a site responsible for higher nervous functions. Moreover, the reports have focused on pituitary imaging and not a comprehensive quantitative analysis of the entire brain.

Recently, there has been an increasing emphasis on detailed investigations of changes in brain morphology as a means of enhancing our understanding of neurological disorders (Shiohama et al., 2019a,b, 2020). This is particularly relevant in the case of CPP because female patients with CPP exhibit a heightened vulnerability to psychiatric disorders in comparison to typically developing children. Our hypothesis posited that this association might stem from the early and excessive exposure of the brain to sex hormones, resulting in distinct structural modifications within the cerebral cortex that deviate from the normative trajectory of brain maturation. To substantiate this hypothesis, our investigation aimed to comprehensively examine and characterize the structural aspects of the brains of CPP patients, utilizing region- and surface-based measurements obtained through advanced structural MRI techniques.

## 2. Methods

### 2.1. Patients

We assembled a sample of 15 female patients with CPP from electronic medical records between 2016 and 2020 at Chiba University Hospital, a tertiary medical center. The clinical diagnoses of CPP for all patients in our sample were confirmed by a certified pediatric endocrinologist using the clinical course and GnRH stimulation tests. Patients with intracranial tumors and those on other mediations, including growth hormone, were excluded. Because gender and age differences were critical issues relevant to the design of this study, we focused our analyses on the brain morphology of girls aged between 6 and 12 years at the time of MRI scan. We obtained the medical records and MRI datasets for all participants. We also identified 13 age-matched girls from our database as NC. These were selected on the basis of age and gender and the absence of neurological disorders, neuropsychological disorders, or epilepsy. Both the CPP and NC MRI datasets were acquired at Chiba university Hospital on the same suite of MRI scanners. The main reason for MRI examination in NC was to rule out intracranial pathologies (*n* = 5/13, 38%), headaches (*n* = 3/13, 23%), orthostatic dysregulation (*n* = 3/13, 23%), and sleep problem (*n* = 2/13, 15%).

### 2.2. Structural MRI acquisition and processing

Three-dimensional (3D) spoiled gradient-echo T1-weighted sagittal images (repetition time/echo time, 7–22/2–5 ms, slice thickness, 1.0–1.4 mm, voxel size 0.5 × 0.5 × 1 mm, matrix 256 × 256) were obtained from all participants included in this study using clinical 1.5 T MRI scanners (GE Signa HDxT 1.5 T, GE Healthcare, Milwaukee, WI, United States). DICOM files were analyzed with the CIVET v. 2.1.0 pipeline (Zijdenbos et al., 2002) on the Canadian Brain Imaging Research (CBRAIN) platform (Sherif et al., 2014). Corrections for nonuniform intensity artifacts were made using the N3 algorithm (Sled et al., 1998), stereotaxic registration (onto the icbm152 nonlinear 2009 template; Fonov et al., 2009), and brain masking (Smith, 2002). Voxel-based volumetric analysis was performed with tissue classification using an artificial neural network classifier (INSECT; Tohka et al., 2004), and segmentation of brain regions was performed with Automatic Nonlinear Image Matching and Anatomical Labeling (ANIMAL; Collins et al., 1999). For our surface analysis, the surfaces of gray and white matter were extracted using 40,962 vertices per hemisphere with the t-Laplace metric (Kim et al., 2005; Boucher et al., 2009). Cortical surface parameters, including the gyrification index (GI), average cortex thickness, cortical surface area, and cortical volumes, were calculated for each hemisphere. Desikan–Killiany–Tourville surface parcellation was used for registration to the anatomical regions. The output of the CIVET pipeline (brain mask shapes, linear and nonlinear registration to the template, tissue classification, and brain segmentation) was manually inspected for quality.

### 2.3. Statistical analyses

Each structural measurement was evaluated using Levene’s test for equality of variances, a two-tailed unpaired t-test for equality of means, and Cohen’s *d*. Cohen’s *d* = 0.8 was recognized as the cutoff value for large size effects. False discovery rate correction for multiple comparisons was performed using the Benjamini–Hochberg procedure (Benjamini et al., 2001; Reiner et al., 2003). Benjamini–Hochberg critical values (*q* = 0.15) were determined for 241 repeating t-tests as *p* < 0.025 in both surface- and voxel-based measurements. SPSS v. 28 (IBM Corp. Armonk, NY, United States) software was used for all statistical analyses. Regional cortical thicknesses were statistically analyzed and visualized as t-statistical maps, and random field theory (RFT) maps using the SurfStat toolbox^1^ with MATLAB R2016a (MathWorks, Natick, MA, USA). The thresholded *p-*value for RFT at cluster-level was determined as 0.02.

## 3. Results

### 3.1. Participants’ background

Table 1 shows the relevant characteristics of both the 15 CPP participants and the 13 NC. In fact, no one with brain tumors and seizures who fit the exclusion criteria was included in the current study. In addition, there were no intellectual disabilities, ASD, ADHD, or epilepsy. All participants were female and born at full-term gestation. Participants’ ages at the time of their MRI scans were not significantly different (*T* (14) = −1.63, *p* = 0.126) between the CPP (*N* = 15) and the NC (*N* = 13) (mean ± standard deviation = 9.3 ± 1.0 and 10.7 ± 3.1 years old in CPP and NC participants, respectively). Qualitative analyses of the brain MRIs showed no abnormal parenchymal findings in either the CPP or NC participants. The mean LH and FSH peaks in patients with CPP, as determined by luteinizing hormone-releasing hormone tests, were 32.6 and 14.1 mIU/ml, respectively. The mean basal level of estradiol was 16.5 pg./mL. These fulfilled the criteria for puberty (Tanaka et al., 2005).

**Table 1:** Demographics and clinical characteristics CPP (*N* = 15).

Characteristics	CPP (N = 15)	NC (N = 13)	*p*
Age (years)	9.3 (1.0)	10.7 (3.1)	0.13
Height SDS	1.8 (1.2)	0.0 (1.0)	<0.01
BMI SDS	0.9 (0.7)	0.2 (0.8)	0.02
Estradiol (pg/mL)	16.5 (14.9)	N.A.	N.A.
<LH-RH test>			
LH peak (mIU/mL)	32.4 (30.5)	N.A.	N.A.
FSH peak (mIU/mL)	13.8 (13.6)	N.A.	N.A.

Mean (Standard Deviation), Student’s *t*-test was used to compare the means between two groups. BMI, Body mass index; CPP, Central precocious puberty; FSH, Follicle-stimulating hormone; LH, Luteinizing hormone;LH-RH, Luteinizing hormone-releasing hormone; SDS, Standard Deviation Score.

### 3.2. Global brain measurements

Cortical volume, surface area, cortical thickness, and GIs showed no significant differences between the CPP and NC participants, except for decreased global white matter volume in CPP participants (*p* = 0.013, Cohen’s *d* = 1.01; Table 2).

**Table 2:** The global brain volume and cortical surface measurements in CPP and NC participants.

	CPP (*N* = 15) Mean [SD]	NC (*N* = 13) Mean [SD]	The rate of CPP/NC	*p*	Absolute Cohen’s *d*
CSF (mm^3^)	37,381 [6536]	40,730 [7127]	0.92	0.21	0.49
Cortical GM (mm^3^)	608,940 [40030]	579,491 [52639]	1.05	0.11	0.64
WM (mm^3^)	371,446 [39881]	422,482 [60952]	0.88	0.013	1.01
Subcortical GM (mm^3^)	36,919 [3133]	37,118 [3948]	0.99	0.88	0.06
Gyrification Index	3.62 [0.10]	3.65 [0.14]	0.99	0.56	0.22
L gyrification index	2.65 [0.06]	2.67 [0.11]	0.99	0.62	0.19
R gyrification index	2.68 [0.07]	2.68 [0.12]	1.00	0.98	0.01
L cortex average thickness (mm)	3.08 [0.07]	2.96 [0.21]	0.97	0.068	0.79
R cortex average thickness (mm)	3.11[0.09]	2.95 [0.22]	0.97	0.025	0.99
L cortex surface area (mm^2^)	94,162 [6076]	97,567 [6702]	1.04	0.17	0.53
R cortex surface area (mm^2^)	94,898 [6238]	97,688 [7288]	1.05	0.28	0.41
L cortex volume (mm^3^)	278,687 [19224]	275,209 [17279]	1.01	0.62	0.19
R cortex volume (mm^3^)	283,489 [17947]	276,445 [16779]	1.03	0.30	0.4

Bold indicates statistically significant. CS, CHARGE syndrome; NC, Normal control; SD, standard deviation; L, left hemisphere; R, right hemisphere; GM, gray matter; WM, white matter; CSF, cerebrospinal fluid

### 3.3. Voxel- and surface-based cortical analyses

The surface-based analyses found no significant differences between the CPP and NC groups in the surface area, thickness, volume, or GI of the cerebrum (Tables 3, 4).

**Table 3:** The candidate measurements using *t*-test in CPP and NC participants.

Category	Measurements	CPP (*N* = 15) Mean [SD]	NC (*N* = 13) Mean [SD]	The rate of CPP/NC	*p*	Absolute Cohen’s *d*
Global	WM (mm^3^)	371,446 [39881]	422,482 [60952]	0.88	0.013	1.01
ANIMAL	Rt frontal GM (mm^3^)	145,253 [9290]	135,683 [115134]	1.07	0.022	0.92
ANIMAL	Lt occipital GM (mm^3^)	41,405 [4686]	36,521 [5054]	1.13	0.013	1.0
ANIMAL	Rt occipital GM (mm^3^)	41,345 [3635]	35,759 [6347]	1.16	0.012	1.1
ANIMAL	Lt frontal WM (mm^3^)	75,111 [8208]	84,753 [12318]	0.89	0.0205	0.94
ANIMAL	Lt temporal WM (mm^3^)	40,170 [3916]	45,949 [7037]	0.87	0.017	1.04
ANIMAL	Lt occipital WM (mm^3^)	17,651 [3186]	21,491 [4797]	0.82	0.018	0.96
Area	Rt Transverse Temporal (mm^2^)	541 [55]	608 [80]	0.89	0.019	0.99
Area	Rt Superior Temporal (mm^2^)	4,954 [414]	5,391 [526]	0.92	0.021	0.93
Thickness	Lt Caudal Middle Frontal (mm)	3.27 [0.08]	3.08 [0.25]	1.06	0.018	1.08
Thickness	Lt Pericalcarine (mm)	2.76 [0.10]	2.53 [0.28]	1.09	0.018	1.07
Thickness	Lt Cuneus (mm)	2.84 [0.08]	2.67 [0.22]	1.06	0.02	1.04
Thickness	Lt Isthmus Cingulate Gyrus (mm)	2.83 [0.09]	2.63 [0.26]	1.07	0.022	1.03
Thickness	Lt Inferior Ocippital Cortex (mm)	2.85 [0.08]	2.69 [0.22]	1.06	0.0245	1.00
Thickness	Lt Precuneus (mm)	3.20 [0.07]	3.00 [0.23]	1.07	0.0083	1.23
Thickness	Rt Caudal Middle Frontal (mm)	3.26 [0.10]	3.05 [0.28]	1.07	0.021	1.04
Thickness	Rt Post Central Gyrus (mm)	2.90 [0.11]	2.73 [0.21]	1.06	0.021	0.99
Thickness	Rt Lateral Orbitofrontal (mm)	3.38 [0.12]	3.14 [0.28]	1.08	0.01	1.16
Thickness	Rt Pericalcarine (mm)	2.78 [0.10]	2.55 [0.27]	1.09	0.012	1.14
Thickness	Rt Paracentral Gyrus (mm)	3.09 [0.15]	2.88 [0.24]	1.07	0.015	1.05
Thickness	Rt Medial Orbitfrontal (mm)	3.16 [0.13]	2.96 [0.25]	1.07	0.019	1.02
Thickness	Rt Cuneus (mm)	2.88 [0.07]	2.73 [0.18]	1.05	0.016	1.09
Thickness	Rt Rostral Middle Frontal (mm)	3.30 [0.11]	3.08 [0.27]	1.07	0.015	1.09
Thickness	Rt Isthmus Cingulate Gyrus (mm)	2.83 [0.10]	2.62 [0.20]	1.08	0.0031	1.36
Thickness	Rt Inferior Occipital Cortex (mm)	2.94 [0.12]	2.77 [0.20]	1.06	0.015	1.04
Thickness	Rt Lingual Gyrus (mm)	2.98 [0.08]	2.76 [0.23]	1.08	0.0065	1.28
Thickness	Rt Lateral Frontal Opercularis (mm)	3.28 [0.10]	3.13 [0.18]	1.05	0.02	1.00
Thickness	Rt Fusiform Gyrus (mm)	3.30 [0.16]	3.11 [0.23]	1.06	0.016	0.98
Thickness	Rt Precuneus (mm)	3.23 [0.07]	3.03 [0.19]	1.07	0.0032	1.42
Thickness	Rt Inferior Parietal (mm)	3.24 [0.12]	3.07 [0.22]	1.05	0.024	0.97
Volume	Rt Lateral Frontal Oribitalis (mm^3^)	2,667 [276]	2,433 [189]	1.1	0.016	0.97
Volume	Rt Pericalcarine (mm^3^)	4,093 [496]	3,629 [499]	1.13	0.021	0.93
Volume	Rt Lingual Gyrus (mm^3^)	7,878 [838]	7,122 [832]	1.11	0.024	0.91

CS, CHARGE syndrome; NC, Normal control; SD, standard deviation; L, left hemisphere; R, right hemisphere; GM, gray matter; WM, white matter; CSF, cerebrospinal fluid. Benjamini-Hochberg critical values (*q* = 0.15) for 241 repeating t-tests was determined as *p* < 0.025.

**Table 4:** The effects of covariates on candidate brain morphologic measurements; univariate general linear model.

Category	Measurements	Adjusted *R* square	Corrected model	The presence of CPP	Age
Global	WM (mm^3^)	0.295	*F* = 1.5, *p* = 0.43	*F* = 2.4, *p* = 0.22	*F* = 1.2, *p* = 0.52
ANIMAL	Rt frontal GM (mm^3^)	0.433	*F* = 1.9, *p* = 0.34	*F* = 1.8, *p* = 0.27	*F* = 1.6, *p* = 0.405
ANIMAL	Lt occipital GM (mm^3^)	0.954	***F* = 24.3, *p* = 0.011**	*F* = 2.2, *p* = 0.23	***F* = 19.9, *p* = 0.015**
ANIMAL	Rt occipital GM (mm^3^)	0.939	***F* = 18.5, *p* = 0.017**	*F* = 0.74, *p* = 0.45	***F* = 14.5, *p* = 0.024**
ANIMAL	Lt frontal WM (mm^3^)	0.209	*F* = 1.3, *p* = 0.48	*F* = 2.48, *p* = 0.21	*F* = 1.1, *p* = 0.56
ANIMAL	Lt temporal WM (mm^3^)	−0.110	*F* = 1, *p* = 0.6	*F* = 3.23, *p* = 0.17	*F* = 0.8, *p* = 0.70
ANIMAL	Lt occipital WM (mm^3^)	0.626	*F* = 2.9, *p* = 0.21	*F* = 0.69, *p* = 0.47	*F* = 2.4, *p* = 0.26
Area	Rt Transverse Temporal (mm^2^)	0.065	F = 1.1, *p* = 0.56	*F* = 0.22, *p* = 0.67	*F* = 0.9, *p* = 0.65
Area	Rt Superior Temporal (mm^2^)	0.169	F = 1.2, *p* = 0.50	*F* = 0.63, *p* = 0.49	*F* = 1, *p* = 0.58
Thickness	Lt Caudal Middle Frontal (mm)	0.673	*F* = 3.3, *p* = 0.18	*F* = 0.38, *p* = 0.58	*F* = 2.6, *p* = 0.23
Thickness	Lt Pericalcarine (mm)	0.588	F = 2.6, *p* = 0.23	*F* = 0.36, *p* = 0.59	*F* = 2.1, *p* = 0.31
Thickness	Lt Cuneus (mm)	0.870	*F* = 8.5, *p* = 0.051	*F* = 0.06, *p* = 0.82	*F* = 6.9, *p* = 0.069
Thickness	Lt Isthmus Cingulate Gyrus (mm)	0.915	***F* = 13.0, *p* = 0.028**	F = 1.9, p = 0.26	***F* = 10.6, *p* = 0.038**
Thickness	Lt Inferior Ocippital Cortex (mm)	0.635	*F* = 3.0, *p* = 0.20	*F* = 0.038, *p* = 0.86	F = 2.4, p = 0.26
Thickness	Lt Precuneus (mm)	0.868	*F* = 8.4, *p* = 0.052	*F* = 0.18, p = 0.70	*F* = 6.2, *p* = 0.079
Thickness	Rt Caudal Middle Frontal (mm)	0.808	*F* = 5.7, *p* = 0.087	*F* = 0.0045, *p* = 0.95	*F* = 4.6, p = 0.12
Thickness	Rt Post Central Gyrus (mm)	0.510	F = 2.2, *p* = 0.29	*F* = 0.16, *p* = 0.71	*F* = 1.8, *p* = 0.36
Thickness	Rt Lateral Orbitofrontal (mm)	0.703	*F* = 3.7, *p* = 0.16	*F* = 0.054, *p* = 0.83	*F* = 2.8, p = 0.22
Thickness	Rt Pericalcarine (mm)	0.808	*F* = 5.7, *p* = 0.088	*F* = 2, *p* = 0.26	*F* = 4.4, *p* = 0.12
Thickness	Rt Paracentral Gyrus (mm)	0.958	***F* = 26.4, *p* = 0.01**	*F* = 0.23, *p* = 0.66	***F* = 21.2, *p* = 0.014**
Thickness	Rt Medial Orbitfrontal (mm)	0.473	*F* = 2.0, *p* = 0.30	*F* = 0.004, *p* = 0.96	*F* = 1.6, *p* = 0.39
Thickness	Rt Cuneus (mm)	0.713	*F* = 3.8, *p* = 0.15	*F* = 0.035, *p* = 0.86	*F* = 3.0, *p* = 0.20
Thickness	Rt Rostral Middle Frontal (mm)	0.750	*F* = 4.4, *p* = 0.12	*F* = 0.007, *p* = 0.94	*F* = 3.4, *p* = 0.17
Thickness	Rt Isthmus Cingulate Gyrus (mm)	0.891	***F* = 10.2, *p* = 0.039**	*F* = 0.86, *p* = 0.42	*F* = 7.1, *p* = 0.066
Thickness	Rt Inferior Occipital Cortex (mm)	0.373	*F* = 1.7, *p* = 0.38	*F* = 0.25, *p* = 0.65	*F* = 1.3, *p* = 0.47
Thickness	Rt Lingual Gyrus (mm)	0.898	***F* = 10.9, *p* = 0.036**	*F* = 4.5, *p* = 0.13	*F* = 7.9, *p* = 0.057
Thickness	Rt Lateral Frontal Opercularis (mm)	0.245	*F* = 1.4, *p* = 0.46	*F* = 0.39, *p* = 0.57	F = 1.1, *p* = 0.55
Thickness	Rt Fusiform Gyrus (mm)	0.210	*F* = 1.3, *p* = 0.48	*F* = 0.47, *p* = 0.54	*F* = 1.05, *p* = 0.57
Thickness	Rt Precuneus (mm)	0.967	***F* = 33.5, *p* = 0.007**	***F* = 10.2, *p* = 0.0497**	***F* = 22.7, *p* = 0.013**
Thickness	Rt Inferior Parietal (mm)	0.327	*F* = 1.6, *p* = 0.41	*F* = 0.003, *p* = 0.96	*F* = 1.3, *p* = 0.49
Volume	Rt Lateral Frontal Oribitalis (mm^3^)	−1.112	*F* = 0.4, *p* = 0.91	*F* = 0.26, *p* = 0.64	*F* = 0.3, *p* = 0.96
Volume	Rt Pericalcarine (mm^3^)	0.345	*F* = 1.6, *p* = 0.40	*F* = 1.3, *p* = 0.34	*F* = 1.3, *p* = 0.47
Volume	Rt Lingual Gyrus (mm^3^)	0.505	*F* = 2.2, *p* = 0.29	*F* = 2.04, *p* = 0.25	*F* = 1.8, *p* = 0.35

Bold indicates statistically significant. CPP, Central precocious puberty; NC, Normal control; SD, standard deviation; L, left hemisphere; R, right hemisphere; GM, gray matter; WM, white matter; CSF, cerebrospinal fluid.

**Figure 1:**
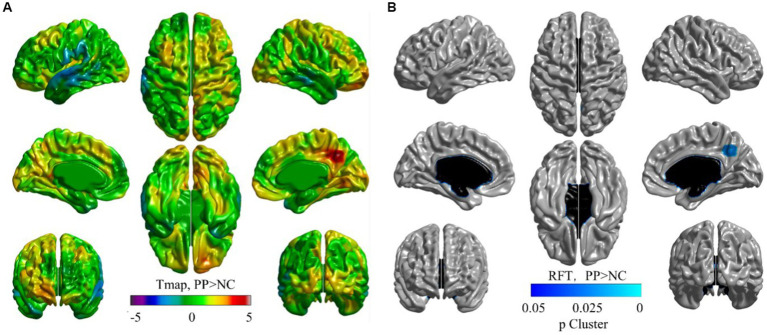
Visualized cortical thickness with a t-statistical map (tmap) **(A)**, and a random field theory (RFT) map (**B**, *p* < 0.02) showing thicker lesions in patients with central precocious puberty (CPP, *N* = 15) than non-CPP controls (NC, *N* = 13). **(A)** The blue color indicates lower mean cortical thickness and the red color indicates higher mean cortical thickness in CPP participants than that in the controls. **(B)** Blue regions denote areas in which CPP participants had significantly thicker cortex at the cluster level than NC. The thresholded *p-*value for RFT at cluster-level was determined as 0.02.

**Figure 2:**
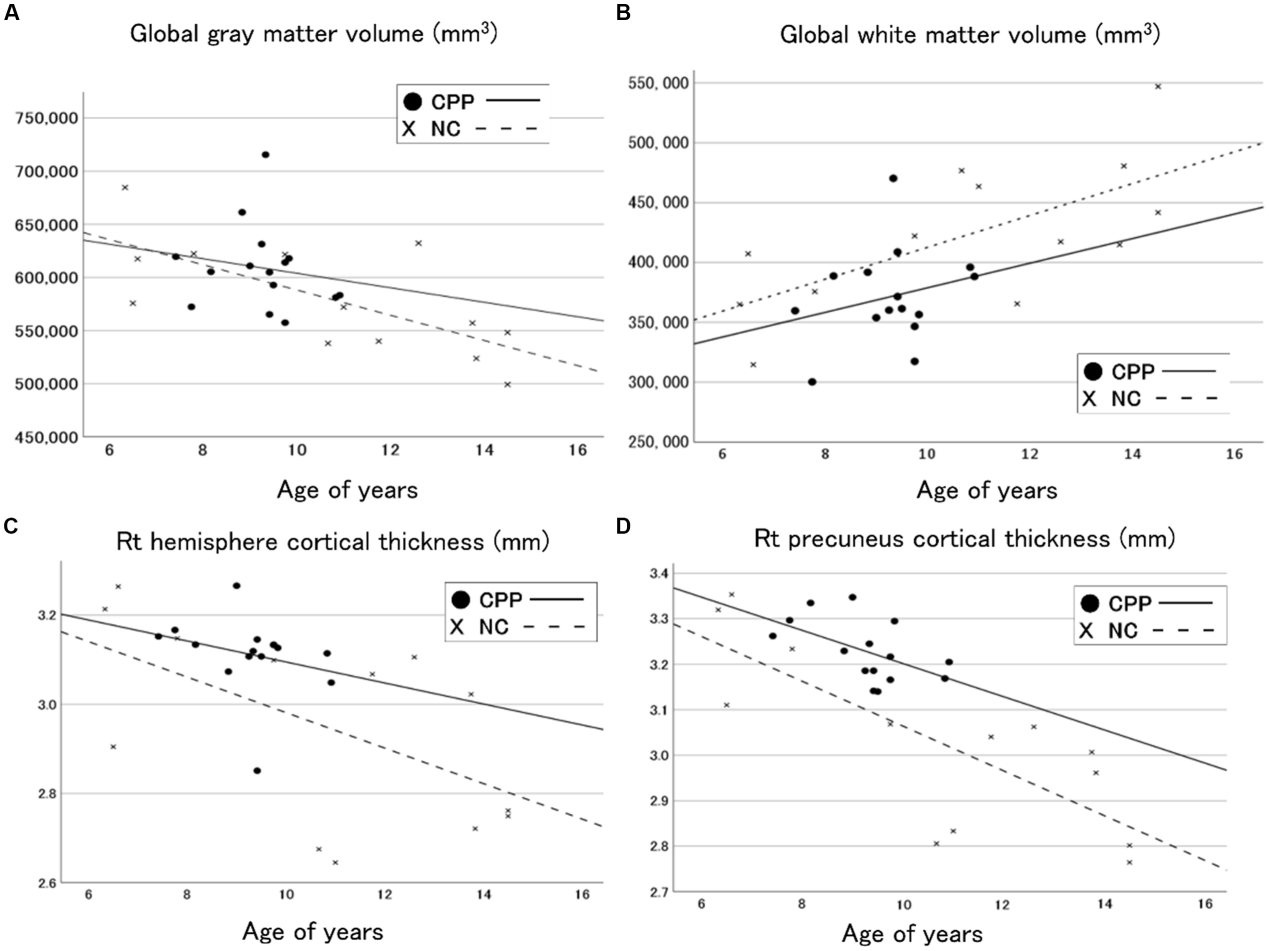
Scatter plots and regression lines (between age and volume) for the global gray matter volume **(A)**, the global white matter **(B)**, the right hemisphere cortical thickness **(C)**, and the right precuneus cortical thickness **(D)** in CPP (closed circle and solid line) and NC (x and dotted line) participants. CPP, central precocious puberty; NC, non-CPP controls; Rt, right.

Figure 1 shows a cortical thickness map superimposed on a 3D brain surface template. *T*-tests showed increased thickness in the right precuneus and decreased thickness in the left superior temporal cortex in the CPP group (Figure 1A). After correction for age with RFT (*p* < 0.02), the right precuneus region was confirmed as significantly different between the two groups in the cortical surface maps (Figure 1B), but there were no significantly thinner regions in the CPP group than the NC group (not shown). Scatter plots showed age-dependent downward trends in global gray matter volume, global cortical thickness, and cortical thickness of the right precuneus in both the CPP and NC groups. Conversely, global white matter exhibited an age-dependent upward trend in both groups (Figure 2).

## 4. Discussion

We analyzed surface and voxel measurements from structural brain MRI scans of patients with CPP and age-matched controls. Thorough regional analyses and surface cortical maps found that the cortical thickness of the precuneus area of the right hemisphere was significantly higher in the CPP than in the NC group. The volume of the right precuneus and global cortical thickness showed age-dependent downward trends in both groups, suggesting the increased thickness of the right precuneus in the CPP group was not simply due to the acceleration of global brain development resulting from early sexual hormone increases. A previous observation study reported that the thinner right rostral middle frontal cortex in the CPP group as compared with that in the NC was a distinct feature of CPP (Yang et al., 2019).

The precuneus is located in the posteromedial cortex of the parietal lobe, which is numerous complex tasks, including visuospatial processing, episodic memory recall, and self-processing operation, i.e., the sense of volition (Cavanna and Trimble, 2006). The precuneus is also involved in the network governing consciousness of the self (Cavanna and Trimble, 2006; Cheng et al., 2018). Puberty is a crucial period for the development of neurocognitive functions (Laube et al., 2020) and the functions of the precuneus are affected by age in adolescents (Killanin et al., 2020). The specific alterations to the precuneus seen in patients with CPP in this study may be related to some of the functions under its control, which show rapid development during puberty. Cortical thickness in the precuneus tends to thin with age in normal adolescent females (Levman et al., 2017), while our findings indicate a thicker cortex in CPP. This suggests that age-related maturation of precuneus function may be affected or altered in CPP. A previous study revealed differences in the brain architecture between CPP and NC in the anterior, middle frontal lobe, with decreased cortical thickness in that region in patients with CPP. The same study also found a significant correlation between cortical thickness and estrogen levels on the basis of surface-based measurements (Yang et al., 2019). However, our study did not see any substantial differences between groups in the cortical thicknesses, volumes, and surface areas of the frontal cortex. The ages of participants may explain this disparity. Patients in our study investigated were older and further into puberty. Our data may indicate features that appear later in puberty in patients with CPP than those identified in the previous report. Animal studies have shown that prolonged exposure to higher levels of estrogen can affect neurons differently from short-term exposure (Woolley, 2007). Our study indicates that other frontal area thicknesses can be indicative of CPP.

Regarding mechanisms, estrogen is a candidate that induces alterations in brain morphology, such as those observed in our study. CPP causes activation of the hypothalamus–pituitary–adrenal (HPA) axis at an inappropriately early age. Increased estrogen levels are an outcome of this and induce the pubescent changes observed in female patients with CPP. GnRH stimulation tests of our patients with CPP verified both increased estrogen level increases and HPA axis activation. Estrogen receptors are expressed throughout the body, including the synapses of the prefrontal cortex. Nevertheless, the effects of estrogen on neurons are debated. Some studies have reported adverse effects on gray matter (Herting et al., 2014; Brouwer et al., 2015), leading to decreased prefrontal cortex volume. By contrast, other research has shown estrogen to have favorable effects on the survival of neurons, the development of dendritic spines, and the formation of synapses (Romeo, 2003). In the present study, the correlation between estrogen levels in the LH-RH test and right precuneus thickness in CPP was 0.17, which was not statistically significant. Estrogen levels exhibit diurnal and daily variations, making it challenging to establish a clear relationship with a single value. Hence, the fundamental mechanisms involved in estrogen-induced alterations to brain morphology require further investigation.

There were several limitations to this study. First, the number of participants was limited. However, patients had similar CPP etiologies and ages of onset. This homogeneity between patients facilitated a detailed evaluation of brain morphology. Further analysis with a larger sample of patients will be required to confirm our results. Second, since this is a cross-sectional study, it does not allow us to infer causal effects. Finally, no psychological scales were administered to our patients with CPP to reveal whether the alterations in brain morphology related to the altered moods and sensitivity observed in patients with CPP. Further prospective studies are warranted to elucidate the effects of brain structure changes in CPP on psychological development. Despite its limitations, we think that this study adds to our understanding of the effects of CPP on brain structure.

## 5. Conclusion

Patients with CPP showed specific morphological brain changes compared with NC. Sex steroids may be involved in the observed structural brain differences in patients with CPP. The features of brain morphology in CPP identified in this study could contribute to further understanding the association between CPP and detrimental psychological outcomes.

## Data availability statement

The raw data supporting the conclusions of this article will be made available by the authors, without undue reservation.

## Ethics statement

The studies involving human participants were reviewed and approved by Chiba University Hospital’s Institutional Review Board. Written informed consent to participate in this study was provided by the participants’ legal guardian/next of kin.

## Author contributions

SY: conceptualization, methodology, writing—original draft, and writing—review and editing. TT: conceptualization, methodology, writing—original draft, formal analysis, data curation, validation, and writing review and editing. TS: conceptualization, methodology, formal analysis, data curation, validation, and writing—review and editing. RT and YK: data curation, validation, and writing—review and editing. SH, HY, and HH: writing—review and editing. All authors contributed to the article and approved the submitted version.

## Funding

This study was supported by the Foundation for Growth Science in Japan, the Japan Society for the Promotion of Science (grant number KAKEN JP21K07694), National Center of Neurology and Psychiatry [grant number: Intramural Research Grant for Neurological and Psychiatric Disorders (3–10)], and Japan Agency for Medical Research and Development (grant number: the Practical Research Project for Rare/Intractable Diseases JP21ek0109498).

## Conflict of interest

The authors declare that the research was conducted in the absence of any commercial or financial relationships that could be construed as a potential conflict of interest.

## Publisher’s note

All claims expressed in this article are solely those of the authors and do not necessarily represent those of their affiliated organizations, or those of the publisher, the editors and the reviewers. Any product that may be evaluated in this article, or claim that may be made by its manufacturer, is not guaranteed or endorsed by the publisher.

## Footnotes

^1^ http://www.math.mcgill.ca/keith/surfstat/

## Abbreviations

ANIMAL: Automatic Nonlinear Image Matching and Anatomical Labeling
CBRAIN: Canadian Brain Imaging Research
CPP: Central precocious puberty
FSH: Follicle-stimulating hormone
GI: Gyrification index
GnRH: Gonadotropin-releasing hormone
HPA: Hypothalamus–Pituitary–Adrenal
LH: Luteinizing hormones
MRI: Magnetic resonance imaging
NC: Non-CPP controls
RFT: Random field theory
RH: Right hemisphere
WM: White Matter
